# Heart Transplantation

**DOI:** 10.3390/jcm13020558

**Published:** 2024-01-18

**Authors:** Nikolaos Chrysakis, Dimitrios E. Magouliotis, Kyriakos Spiliopoulos, Thanos Athanasiou, Alexandros Briasoulis, Filippos Triposkiadis, John Skoularigis, Andrew Xanthopoulos

**Affiliations:** 1 Department of Cardiology, University Hospital of Larissa, 41110 Larissa, Greece; nikoschrisakis8@yahoo.gr (N.C.); ftriposkiadis@gmail.com (F.T.);; 2 Department of Surgery, University Hospital of Larissa, 41110 Larissa, Greecespiliopoulos@med.uth.gr (K.S.); t.athanasiou@imperial.ac.uk (T.A.); 3 Department of Clinical Therapeutics, Faculty of Medicine, Alexandra Hospital, National and Kapodistrian University of Athens, 11528 Athens, Greece

**Keywords:** heart transplantation, heart failure, indications, contraindications, complications, xenotransplantation

## Abstract

Heart transplantation (HTx) remains the last therapeutic resort for patients with advanced heart failure. The present work is a clinically focused review discussing current issues in heart transplantation. Several factors have been associated with the outcome of HTx, such as ABO and HLA compatibility, graft size, ischemic time, age, infections, and the cause of death, as well as imaging and laboratory tests. In 2018, UNOS changed the organ allocation policy for HTx. The aim of this change was to prioritize patients with a more severe clinical condition resulting in a reduction in mortality of people on the waiting list. Advanced heart failure and resistant angina are among the main indications of HTx, whereas active infection, peripheral vascular disease, malignancies, and increased body mass index (BMI) are important contraindications. The main complications of HTx include graft rejection, graft angiopathy, primary graft failure, infection, neoplasms, and retransplantation. Recent advances in the field of HTx include the first two porcine-to-human xenotransplantations, the inclusion of hepatitis C donors, donation after circulatory death, novel monitoring for acute cellular rejection and antibody-mediated rejection, and advances in donor heart preservation and transportation. Lastly, novel immunosuppression therapies such as daratumumab, belatacept, IL 6 directed therapy, and IgG endopeptidase have shown promising results.

## 1. Introduction

Heart transplantation (HTx) is the final treatment for patients with advanced heart failure [1]. The first attempt was made 118 years ago, in 1905, by Alexis Carrel and Charles Guthrie at the University of Chicago, and the second attempt was made by Mann in 1933, both in dogs. Twenty years later, Marcus at the Chicago Medical School studied methods of preserving grafts while developing techniques to make the graft act as a pump. The next great scientist was Demikhov; from 1951 to 1962, he laid the foundations for heterotopic HTx with experiments in dogs. In 1953, Neptune applied hypothermia to the recipient and donor. In 1957, Webb and Howard applied the preservation of grafts to potassium solutions, and in 1959, Goldberg at the University of Maryland performed the first orthotopic HTx in dogs. The same year, Cass and Brock at the Guy’s Hospital in London performed autotransplantation and homologous transplantation and essentially introduced the bicaval technique, while in 1950, Dr. Shumway at Stanford performed orthotopic HTx in dogs with cardiopulmonary bypass. In 1960, as the concept of graft rejection was beginning to be understood, Lower achieved a recipient dog lifespan of more than 6 months with the administration of corticosteroids and azathioprine. In 1964, Hardy at the University Hospital in Jackson, Mississippi, performed the first human transplantation (xenotransplant) using a chimpanzee heart. Then, in 1967, the first interhuman HTx was performed by Dr. Christiaan Nethling Barnard at Groote Schuur Hospital in Cape Town, South Africa. The donor was a little girl who had been hit by a vehicle by a drunk driver, and the recipient was a 54-year-old patient with end-stage ischemic heart disease who survived 18 days. Since then, significant progress has been made in the field of HTx [2].

The present work is a clinically focused review discussing current challenges and future perspectives in the field of heart transplantation.

## 2. Graft Selection

Whether HTx is successful or not depends on several factors. The most important factors are discussed below.

### 2.1. ABO and HLA Compatibility

ABO blood group antigens are in the membranes of erythrocytes and endothelial cells of tissues. Despite the tolerance shown by the immune system of newborns with transplants incompatible with the ABO system, in adults, ABH antibodies bind to the endothelium of the graft with activation of the complement leading to injury and necrosis of the allograft in the process of hyperacute rejection [3]. Due to the limited number of available grafts, attempts for transplants incompatible with the ABO system have been made. Studies have shown that those performed after 2005 do not show a difference in mortality and the need for retransplantation, suggesting the optimization of immunosuppression protocols in the above cases [4].

HLA (human leucocyte antigens) are glycoproteins of the surface of the cell membrane encoded by genes in the class I and class II regions of the major histocompatibility system on chromosome 6. In the first class, the proteins HLA-A, HLA-C, and HLA-B are found in all cells and recognized by CD8+, and in the second class, the proteins HLA-DQ and HLA-DP are located in antigen-presenting cells and recognized by CD4+ T-helper cells. Risk factors for the production of antibodies against the above are pregnancy, previous transplantation, and the use of circulatory support devices [3]. Due to the lack of grafts, for HTx, in contrast to other organs, compatibility testing is not performed in many centers. Studies have shown that HLA mismatch does not affect mortality but is associated with increased graft vasculopathy [5]. From the above data, the need to administer induction therapy to patients with detected antibodies against HLA is questioned and increases the chances of finding grafts from candidate donors [6].

### 2.2. Graft Size

Probably the most important parameter in graft selection is matching the appropriate graft size to the candidate recipient. Multiple measurements have been used in the past, such as height, body weight, body mass index (BMI), and body surface area (BSA) [7]. Now, in many centers, a relatively new measure called “Predicted Heart Mass (PHS)” is used, which is the sum of the calculated mass of the left and right ventricles. Studies showed that this marker is superior compared to the traditional measurements for the appropriate selection of implants [8] and predicts one-year mortality after transplantation [9]. Another marker that emerges in the literature and seems to correlate well with donor and recipient size matching is called “Predicted Lean Body Mass” (PLBM) [10].

Choosing the right graft size plays an important role in patient survival. Smaller than ideal grafts show chronotropic failure with a need to increase cardiac output. This is achieved by increasing filling pressures at a chronic level with detrimental consequences for the graft and, as a result, for patient survival. On the other hand, grafts larger in size compared to the ideal seem to adapt better to the requirements of the recipient. There is no consensus on the effect of the latter on patient survival. On the one hand, some studies do not find differences in events during hospitalization and in the short-term outcomes [11], while others find an increase in one-year mortality after transplantation [12].

### 2.3. Ischemic Time

The time during which the graft is removed from the donor until it is transplanted into the recipient is called ischemic time, and this plays a very important role in both the viability of the graft and the survival of the recipient. The usual tactic is to transfer the grafts using the static cold storage technique, in which the heart is placed in a cold preservation solution and transferred to a special icebox. However, hypothermia can also have harmful effects as it causes a redistribution of cell membrane lipids, affecting its integrity while diverting its metabolism from aerobic to anaerobic, increasing oxidative stress. The mechanism of damage that the graft is subjected to due to transfer from the donor to recipient is called ischemia/reperfusion injury. During hypoxia, there is a deficit in adenosine triphosphate (ATP) production, leading to dysfunction of Na^+^/Ca^++^ channels, resulting in an increase in the intracellular concentration of Ca^++^ which causes the production of an increased amount of free radicals. At the same time, the endothelium produces vasoconstrictor and pro-inflammatory factors that increase the damage to the ischemic area. On the other hand, during reperfusion, the activation of leukocytes is promoted by the produced cytokines and proteases, while the restoration of the function of the Na^+^/Ca^++^ channels leads to an increased uptake of intracellular Ca^++^. This results in increased free radical production and mitochondrial swelling [13].

In routine practice, the time period required for a graft to be transplanted is divided into routine cold ischemic time (<4 h) and prolonged cold ischemic time (>4 h). In the international literature, a multitude of studies are presented where specific donor characteristics are reported to make the respective grafts more resistant to extracorporeal ischemia. A recent study showed that grafts derived from obese donors subjected to prolonged cold ischemic time reduced the first 30-day mortality and graft failure of recipients while increasing the survival of recipients at 1 and 5 years after transplantation; however, this did not apply to routine ischemic time. The pathophysiological mechanism is hypothesized to lie in increased leptin activation which enhances RISK-NOS pathway signaling, contributing to ischemic graft conditioning [14]. Additionally, in another study, it was observed that grafts from older patients exposed to an ischemic time of less than 120 min had better results compared to younger ones for the same time period [15], and another reported that ischemic time beyond 3 h is associated with increased mortality. Interestingly, there appeared to be greater vulnerability for grafts from blood group O donors compared to the others [16]. Finally, there is preliminary data on the use of special devices where ex vivo non-ischemic heart preservation is carried out to reduce the negative effects of the heart outside the body with very encouraging results [17].

### 2.4. Age

Age plays an important role in donor selection. The upper age limit is 60 years old to ensure better and longer-term function of the graft and reduce mortality after transplantation. Research has found an inversely proportional relationship between donor age and recipient survival. The most likely mechanisms are the increased atherosclerosis to which these grafts are subjected, the greater rate of fibrosis, greater vulnerability to cold ischemic time, and valvular lesions [18]. This is confirmed by studies that showed that groups of elderly patients who received grafts from older donors showed a negative impact on one-year survival [19]. This is contrary to what is happening in the general practice in an attempt to widen the age limits. On the other hand, other studies have shown that grafts from older donors offer longer donor survival when they have undergone a shorter ischemic cold time [15]. Adjustments for donor factors, such as smoking or cause of death and for recipients on ciclosporin administration and age, negated the statistical difference in acute and overall mortality between “younger” and “older” transplants [20]. Therefore, allografts from older donors could be given to older patients who are low on the waitlist or too frail, improving their quality of life [21].

### 2.5. Infections

A very important factor that is strictly controlled during the selection of grafts is the possible infection of the donor. Most centers have protocols in place to screen for a range of potential infections, including HIV, hepatitis B/C, cytomegalovirus (CMV), Epstein–Barr virus (EBV), human T cell leukemia–lymphoma virus, herpetic viruses, systemic viral infections (e.g., measles, rabies, adenovirus, enterovirus, and parvovirus), prion-related disease, and syphilis [7]. The finding of bacteremia is also a contraindication for HTx. Contrary to recent studies, HCV-positive donors are now a new “reservoir” as recipients who developed hepatitis from transplants have very good results with the administration of new specific antiviral drugs, which are well tolerated [22]. Finally, early studies showed no difference in one-month mortality between patients who received grafts from COVID (+) patients compared to COVID (−) [23].

### 2.6. Cause of Death

Cause of death is an important criterion for transplant eligibility. The main “source” is from donors who are victims of circulatory accidents, fatal gunshot wounds without damage to cardiac structures, brain death from anoxic conditions, or histological pathologies of the parenchyma, such as tumors (exceptions are gliomas and medullablastomas). An exception is the rupture of a cerebral aneurysm, as it is a sign of possible serious cardiovascular disease, and the donor is put under thorough control. Additionally, great care is given to patients who end up with carbon monoxide poisoning, as this causes myocardial licking and increases the chances of coronary events and arrhythmias in the future [7]. Finally, there is an increasing tendency to use transplants from circulatory death patients with comparable results from brain death donors [24]; this seems to contribute to a reduction in the waitlist and increases the chances for transplantation for older people and those with more co-morbidities [25].

### 2.7. Laboratory-Imaging Tests

During brain death due to dysautonomy of the autonomic nervous system, hemodynamic instability occurs with parallel electrolyte and acid–base balance disturbances. Many consensus papers recommend targeting a donor’s mean arterial pressure > 65 mmHg with the lowest possible dosage of inotropic and vasoconstrictor drugs. Additionally, if an ejection fraction is found to be <45%, it is re-checked in 6 h, and the donor is put under more extensive control. From biochemical testing, N-terminal pro–B-type natriuretic peptide (NT-proBNP) > 160 pg/mL is associated with reduced cardiac index, troponin shows mixed results, blood urea nitrogen/creatinine > 1 ratio exhibits a negative prognostic significance, and, finally, elevated procalcitonin is an indication of severe donor infection. Rarely, investigation with Swan–Ganz catheterization in an effort to maintain central venous pressure 8–12 mmHg, pulmonary capillary wedge pressure < 14 mmHg, mean arterial pressure > 65 mmHg, and left ventricular function index > 15 gm/m is needed. On the other hand, some studies do not support the correlation of the donors’ pathological hemodynamic values with the recipients’ survival [26]. Finally, in some centers, donors over 40 years of age undergo coronary angiography, and if lesions > 50% are found, the graft is rejected most of the time [7].

## 3. Allocation System—Graft Distribution

In 2018, UNOS renewed the transplantation system for patients on the heart transplant waitlist. The purpose of this was to prioritize patients with a more severe clinical condition in order to achieve a reduction in the mortality of people on the waiting list. These changes were necessitated by the increase in the number of candidate recipients and the increase in the use of mechanical circulatory support devices (MCSD). The 3-tiered system (1A,1B,C) was modified to a 6-tiered system (1–6). Category 1A of the old system, which included patients who could not be discharged from the hospital, was divided into three categories. The 1st refers to patients with MCSD or who are at risk of potentially fatal ventricular arrhythmias. The 2nd category includes patients with an intra-aortic balloon pump (IABP), a non-dischargeable left ventricular assist device (LVAD), other devices with long-term circulatory support, dysfunctional circulatory support devices, or patients experiencing potentially fatal ventricular arrhythmias without circulatory support. The 3rd category includes patients in need of administration of inotropic drugs who need continuous monitoring, with mechanical circulation support after a certain period of time depending on the system, or with systems with complications. Category 1B was divided into a 4th category for patients with LVADs who have been using them for more than one month, retransplants, and patients with cardiomyopathies as well as ischemic heart disease with intractable angina pectoris. Finally, category 2 was divided into two others: the 5th for patients on the list for transplantation for more than one organ, and the 6th for the remaining candidates [18].

This new classification brought about significant changes in the management and distribution of grafts, presenting several positive elements but also receiving a lot of criticism from a large part of the scientific world due to the negative effects it brought simultaneously. As far as the positive elements brought about by the new classification, a reduction in the waiting time of people who were in circulatory shock and were mechanically supported was initially observed. Indeed, data showed that the volume of transplants in the United States of America at the local, state, and national levels did not change, but at the same time, there was a decrease in the waitlist [27]. Also, with the new classification strategy, women had a better chance of transplantation, with no difference compared to men in terms in terms of survival. However, women were less likely to use temporary circulatory support [28]. In addition, the new classification system benefited patients on the waiting list for simultaneous heart and lung transplantation and reduced early post-transplant mortality [29], as well as patients with congenital heart defects [30]. Finally, there is greater geographic sharing following the 2018 heart allocation policy [31].

On the other hand, several disadvantages of the new system emerge from the literature review. Initially, studies show that patients with a very serious condition now have better chances for a transplant, but for the rest who undergo a transplant, there is no significant difference [32]. Additionally, in the new system, the distance between the graft and transplant center should be between 500 miles for group 1 and 2 patients and up to 250 for group 3. This new classification has substantially increased the distance compared to the old one, aiming to increase the probability of finding a suitable graft. However, this way, the ischemic time is increased significantly, while the disparities between the geographical areas and the patients who live in them remain [33]. Another disadvantage is the fact that after the reclassification, there has been an increase in the use of temporary circulatory support devices. However, studies have reported significant variation in the use of these devices between centers since there is no uniform protocol. Therefore, questions are raised about the possible existence of inequality among patients as far as the possibility of transplantation [34]. In addition, with the new system, priority is given to patients who are in a more critical condition. However, the survival rate of patients after transplantation and their quality of life are not taken into account. Additionally, the increased use of temporary devices potentially causes several complications, causing damage to other vital organs of the patients and reducing their chance of survival [35]. Post-transplant renal failure requiring dialysis as a complication showed an increase in the new classification system. From an analysis of the data, it appeared that pre-transplant estimated glomerular filtration rate (eGFR) and the use of extracorporeal membrane oxygenation (ECMO) are predisposing factors [36]. Finally, women have a higher rate of exclusion from the waiting list in category 1 because of complications for which no obvious explanation has yet been given [37].

A major change brought about by the new system is the rapid increase in mechanical circulation support. Regarding temporary circulatory support, several studies have been conducted on the most widely used devices. There was an increase in IABP use, which improved the waiting time and survival rates after transplantation, while the possibility of axillary access offers very good results in terms of bridge to recovery, complications, and rehabilitation of patients [38]. At the same time, the data on the use of the Impella are also encouraging. With the new classification system, its use has increased, with patients assigned to it having a shorter waiting time, a higher chance of transplant, and reduced mortality while waiting [39]. It offers very good results in terms of survival and post-transplant complications in patients in whom it has been placed as a bridge to transplant [40]. It is also a very good option for the treatment of cardiogenic shock as a bridge to transplant with low pre- and post-transplant complications [41] and causes unloading of the right ventricle and decongestion. At the same time, there is no correlation between the duration of use of the device and renal failure requiring hemodialysis [42]. Finally, the successful use of Impella in both ventricles at the same time is reported with encouraging results [43]. On the other hand, the use of Impella over IABP did not bring about substantial changes in terms of the waitlist [44].

Extensive reference is also made to VA-ECMO, which shows many positive elements. From studies conducted, VA-ECMO-supported patients enrolled in the new system had increased odds of transplant, lower odds of removal from the waiting list and mortality, and increased 6-month post-transplant survival [45]. Additionally, ECMO had no effect on duration of surgery, hospitalization, and 1-year mortality [46]. In terms of bridging, studies with patients in whom an ECMO bridge to transplant was placed compared to those in whom it was removed did not show differences in mortality [47], while patients on VA-ECMO showed no difference in mortality between bridging to transplant or to LVAD [48]. Finally, it is worth mentioning that a study in Korea showed that ECMO patients waiting for a transplant, as long as they are stable, should maintain the arrangement of their device (central, peripheral), and no attempt should be made to change it if it is not necessary [49].

Nevertheless, limitations of the abovementioned devices have also been reported, which require careful selection of the potential candidate recipients. It was observed that transplant patients who were fitted with temporary circulatory support devices had no difference in mortality compared to those without devices but were more likely to have complications [50]. Spanish registries claim that temporary LVAD had better results than ECMO [51]. In addition, co-morbidities seem to negatively affect the effect of the device. In a study also from Korea, it was shown that patients under ECMO in need of mechanical ventilation have increased pre-operative mortality [52], while patients with a high MELD score under VA-ECMO have increased mortality [53]. Still, the use of temporary circulatory support in the new classification excludes the possibility of myocardial resuscitation of patients to achieve faster transplantation [54]. Finally, during the COVID-19 epidemic, the use of temporary circulation support decreased, with researchers suggesting that patients with a more severe clinical status did not have easy access to care facilities [55].

Interesting features emerge from studying long-term circulatory support devices. Initial data from registries show that in the new system, there is a reduction in the use of LVAD devices and, on the contrary, an increase in short-term circulatory support devices such as VA-ECMO and IABP as a bridging strategy, therefore significantly increasing the length of time patients are hospitalized [32]. In contrast, patients using an LVAD as a temporary bridge to transplant did not show a difference in one-year survival compared to patients who had an LVAD placed for long-term support [56]. Notably, with the new system, patients with LVADs have a lower chance of dying or being excluded from the list due to deterioration. Nevertheless, they show a higher mortality after transplantation and an increased probability of retransplantation [57]. Regarding the comparison of long-term circulatory support and temporary circulatory support devices, it is observed that LVAD had the same postoperative results compared to IABP [58], BiVAD patients had the same outcomes as LVAD patients classified in the second category in the new system [56], whereas the use of the total artificial heart device as bridging from VA-ECMO for patients awaiting transplantation is a viable option for a group of highly selected patients [59]. Patients with LVAD devices used for temporary circulatory support show a survival advantage compared to those with VA-ECMO used for bridging to transplantation [60], while it appears that the combined use of VA-ECMO with IABP in transplant patients did not offer additional benefits in either morbidity or mortality [61].

## 4. Transplantation Techniques

### 4.1. Orthotopic Transplantation

Initially, a thoracotomy is performed once the graft is available in the operating room. The patient is set on bypass, and the aorta is clumped. Then, the superior vena cava is transected at the cavo–atrial junction, the great vessels are transected at the level of their valves, and the left atrium is transected by entering the roof of the left atrium, leaving the orifices of the pulmonary veins. In the next step, the graft is prepared. The large vessels are separated from each other, and the connection of the pulmonary artery to the left atrium is cut in the latter with an incision that joins the orifices of the pulmonary veins, creating an empty opening. The placement of the graft in the recipient can be performed in two ways:

(A) Bicaval technique: The placement of the graft in the recipient is an anastomosis of the left sinus of the graft with part of the recipient. Initially, suture placement is performed at the left atrial cuff adjacent to the left superior pulmonary vein and passing through the donor’s left atrial cuff adjacent to the left atrial appendage. Then, it is performed sequentially or posteriorly, and the anterior suturing of the sinus with care for the surfaces of the endocardium is sutured to reduce the possibility of thrombus formation. After this, the superior and inferior vena cava are anastomosed, taking care not to injure the coronary sinus, pulmonary artery, and aorta.
(B) Biatrial technique: The anastomosis of the atrium is performed as above, with the sutures ending at the inter-atrial septum. The difference is that part of the recipient’s right sinus is also preserved, and an anastomosis is also performed there. The suture is initiated at the superior end of the atrial incision, and the anastomosis of the diaphragm follows. Then, the great vessels are sutured as before. After the anastomoses, the patient is placed in the Trendelenburg position, the cavities are vented, temporary right atrium and ventricle pacing is instituted, and the patient is weaned from mechanical circulation.

### 4.2. Heterotopic HTx

Heterotopic HTx is used in pulmonary hypertension, in smaller grafts or those exposed to increased ischemic time, and in order to support circulation from the recipient’s native heart during severe graft rejection. First, the excess of the descending aorta is resected, and the right pulmonary veins are prepared together with the corresponding lung, while the left ones are prepared individually. Then, the left atrium is resected at the point of the pulmonary veins, and the superior and inferior vena cava are also resected. The heart is then rapidly decompressed with the left superior pulmonary vein, left atrium, and inferior vena cava incisions while the aorta is clubbed and cardioplegic fluid is infused.

In the recipient, we have superior and inferior vena cava and aorta cannulation. An anastomosis is performed on the part of the graft’s left sinus incision and the recipient’s left sinus incision. An end-to-side aorto-aortic anastomosis and an end-to-side donor pulmonary artery to the recipient’s right atrial wall are then performed. Finally, after superior and inferior vena cava anastomosis, air is removed with the patient in the Trendelenburg position.

Studies have shown that bicaval anastomosis is less likely to require a pacemaker, and patients need less time in the hospital [62].

## 5. Transplant Recipients

Transplant recipients should meet certain selection criteria and have no contraindications for HTx. The indications for heart transplantation according to the European Society of Cardiology (ESC) and American Heart Association (AHA) guidelines are listed in Table 1.

There are also specific contraindications for heart transplantation, listed in Table 2.

## 6. Complications

### 6.1. Rejection

Rejection is a serious complication of transplantation. The clinical picture varies from asymptomatic with findings on endomyocardial biopsy to cardiogenic shock. Studies show that patients with microcirculatory resistance index [65], donors with macrophages with activated C-C chemokine receptor 2 (CCR2) and myeloid differentiation primary response protein 88 (MYDD8) [66], increased left ventricle posterior wall thickness (1 mm increases 66%) and increased left ventricle mass index (1 g/m^2^, the chance increases 2.7%) [66,67], have an increased chance of rejection. Three types of rejection have been identified, which are described below.

1. Acute cellular rejection: This is the most frequent type of rejection. Pathophysiologically, the major and minor histocompatibility antigens are not uniformly expressed, with the result that they function as allografts and activate T-cytotoxic lymphocytes either indirectly or through antigen presentation. CD-4 and CD-8 positive T lymphocytes with high affinity to interleukin-2 receptors and increased intercellular adhesion molecules with high MHC-II expression on cardiac myocytes produce cytokines. This leads to the accumulation of inflammatory cells, such as macrophages and neutrophils, perivascularly inducing inflammation in the epicardial and endomyocardial arteries. Histopathologically, it is divided into three categories. The first is called low-grade, where inflammation is not observed in the myocardium. The second is the partial degree, where two or more foci are found in the myocardium. Finally, the third one, identified as high grade, shows multiple foci of damage with various types of inflammatory cells and necrotic elements.
2. Acute humoral rejection: This shows a more complicated clinical manifestation. It is believed that the production of antibodies against the major and minor histocompatibility complex system is induced due to previous exposure to allogens such as transfusion, transplantation, and long-term circulatory support devices. It is divided into five categories according to antibody-mediated rejection in histopathological and immunopathological studies. The first is pAMR 0 Negative for antibody-mediated rejection with negative histopathological and immunopathological studies. The second is called pAMR 1(H+), with the presence of histopathological findings such as activated immune cells, inflammation, necrosis, etc. The third is pAMR 1(I+) antibody-mediated rejection with immunopathological and not histopathological findings. Next is pAMR 2, combining histopathological and immunopathological findings. Last is pAMR 3, in which severe histopathology (hemorrhage, capillary fragmentation, inflammation, interstitial edema) and immunopathological markers are observed.
3. Hyperacute: This is created due to incompatibility with the ABO system, and its manifestations begin early with thrombosis of the vessels of the graft [68].

The main categories of immunosuppressants are:

1. Corticosteroids

These are mainly used in the initial phase of immunosuppression and in acute rejection episodes. They enter the cell’s nucleus and modify the expression of many genes. At an immunological level, they increase the number but reduce the mobility of neutrophils while reducing the production of many factors that stimulate the inflammatory process. Due to their many side effects, their use is usually limited to the first six months after transplantation [69], while their prolonged administration is associated with reduced survival [70].

2. Calcineurins inhibitors

Cyclosporine and tacrolimus are the two drugs used in this class. Their action lies in inhibiting the synthesis of interleukins that activate T-lymphocytes, especially the helpers. A serious side effect is the worsening of kidney function [68]. It has been found that the use of diltiazem helps to maintain cyclosporine levels with lower doses of administration, protecting kidney function [71]. From studies, tacrolimus seems to be superior, especially in terms of side effects [72].

3. Anti-proliferative agents

This category includes azathioprine and mycophenolate. They are purine analogs where, through enzymes, they are converted into metabolites that mimic the action of purines by inhibiting DNA replication. In this way, they inhibit cell division and reduce the robustness of the immune system [68]. These two drugs have a similar effect, with the second tending to replace the first in resistant rejection. It was shown that patients with thiopurine S-methyltransferase gene variants show a reduced benefit compared to the rest [73].

4. mTOR inhibitors

Medicines in this category are sirolimus and everolimus. Inhibition of the mTOR pathway results in the inhibition of several interleukins and reduces T lymphocyte replication, decreased worsening of graft vasculopathy, decreased likelihood of malignancy compared to mycophenolate [74], and the possibility of de-escalation of ciclosporin dose with better results in terms of patient’s renal function outcome, according to the Madela study [75]. Additionally, the combination with tacrolimus appeared to reduce the hypertrophy that can develop in the graft as well as the amount of fibrosis at 1 year [76]. Conversely, an increase in the triglyceride levels of these patients has been observed, leading to the need for regular biochemical control of patients receiving the drug [77].

5. Induction Therapy

Induction therapy is used to introduce immunosuppression, avoid acute rejection, and maintain the other drugs at lower doses. It can also be given in case of relapse of graft rejection. The drug groups are as follows:

- Monoclonal anti-lymphocyte antibodies;
- Polyclonal anti-lymphocyte antibodies;
- Antibodies against cytokine receptors.

Treatment protocols usually include a steroid drug, a calcineurin inhibitor, and usually mycophenolate. Steroids, due to side effects, are discontinued within the first 6 months to 1 year in half of patients, with nearly 90% discontinued at 24 months [78].

### 6.2. Graft Angiopathy

The most common cause of death after 1 year of transplantation is probably of an immunological origin (hypersensitivity reaction), where the T-helper cells are activated by antigens of the endothelial cells, promoting the production of pro-inflammatory substances, which leads to an attack on the smooth muscle fibers of the vessels causing their hyperplasia, resulting in the narrowing of the lumen. There is also evidence of the involvement of natural killer cells. Additional causes of graft vasculopathy include obesity, coronary artery disease, dyslipidemia, diabetes, donor age, male gender, brain-dead donors, increased graft ischemia time [79], hypercholesterolemia [80], increased end-diastolic diameter and decreased interleukin 33 (IL-33), as well as the increased suppression of tumorigenicity 2 (ST2) [81]. Angina is rarely a symptom, as the grafts lack innervation (they are denervated upon removal from the donor) while suffering the same complications as classic coronary artery disease. Its diagnosis is a challenge, as coronary angiography essentially functions as an allography of the vessels, often underestimating the thickening of the vessel walls. A classification has been proposed by the International Society for Heart and Lung Transplantation (ISHLT) that classifies vasculopathy into three categories (Table 3) [82].

The use of intravascular ultrasound (IVUS) significantly increases diagnostic accuracy, while stress echo seems to be gaining ground, as well. Immunosuppression has limited effects, with mycophenolate showing the best results. The gold standard method is still biopsy, which is performed at regular intervals up to 5 years after the transplant, as this specific complication lacks typical symptoms or may be asymptomatic for a long time without a specific widely accepted protocol. Efforts are being made through the use of magnetic resonance imaging (MRI) to avoid complications of the biopsy, which are still in the early stages [83].

### 6.3. Primary Graft Failure

Primary graft failure is the leading cause of death in the 1st month after transplantation. It affects the left and/or right ventricle, with ultrasound findings and hemodynamics ranging from mild manifestations to hemodynamic instability requiring inotropes and mechanical circulatory support. Pathophysiologically, the involvement of several mechanisms is thought to contribute. Initially, a serious role is played by the ischemia time of the graft. Despite being immersed in ice-cold solutions, the graft suffers ischemia with disruption of the Na^+^/K^+^ pump function, resulting in the diversion of metabolism to anaerobic and, at the same time, the appearance of cell swelling. On the other hand, reperfusion of the graft after transplantation leads to the release of free radicals with further deterioration of cellular homeostasis. In addition, the brain death of the donor plays an important role. Due to the increased catecholaminergic activity of the donor, desensitization of β-receptors is caused, while an overload of the cell with Ca^++^ is created, thus contributing to the appearance of myocardial stunning [84].

There are several predisposing factors responsible for this particular complication reflected in the RADIAL score, and they are right atrial pressure > 20 mmHg, donor age > 30 years, recipient age > 60 years, graft ischemia time > 4 h. Others are pre-operative LVAD use, female gender [85,86], obesity [87], and diabetes [88]. When donor risk factors are categorized, they are brain death, use of intravenous inotropes, age, and dysfunction of another donor graft. Contributors include age, fibroin use, mechanical ventilation, circulatory support devices, pulmonary hypertension, obesity, and diabetes. Finally, regarding the procedure, the ischemia time, donor–recipient mismatch, and transplantation from a female donor to a male recipient have been implicated [84]. The ISHLT has set certain criteria for grading the severity of this particular complication (Table 4).

Early right ventricular failure is a serious complication of transplantation. To be diagnosed, acute right heart failure must occur in the absence of pulmonary hypertension, myocardial damage, and graft rejection. The pathophysiological mechanism is similar to that of the left ventricle described above. It usually appears immediately, in the first 24 h after the operation, but can also occur later, with the peak on the 3rd postoperative day, and usually lasts a week. Patients with severe disease also have an increased chance of dialysis. Treatment ranges from inotropic support to mechanical circulatory support [89]. It has been found that filling pressures in the right ventricles usually show normal values at 3–6 months after transplantation, and improvement in their function is expected in the 1st year after the operation [90]. Finally, the measurement of pulmonary arterial elastance shows a strong correlation between mortality in 1 year and the possibility of developing right heart failure, and some authors recommend its regular measurement in transplant patients [91].

Treatment is mainly focused on supporting the graft until it regenerates. Often, there is a need for the use of inotropic drugs, and sometimes, there is also a need for the use of circulation support devices. From studies it has been observed that the use of VA-ECMO is a very good choice. It also appears that the shorter use of ECMO seems to have more benefit in early survival while showing better results than LVAD [92].

### 6.4. Infections

Infections are a common complication of transplantation due to the immunosuppression administered. The most common infection is CMV, followed by EBV, herpes, the adenovirus that causes severe morbidity and mortality with many myocardial complications [93], and bacterial infections. Protozoa and fungi follow these. The most common Gram-negative bacterial infections are extended-spectrum beta-lactamase (ESBL) *Escherichia coli* and *Klebsiella pneumoniae*, *Pseudomonas aeruginosa*, and carbapenem-resistant *Klebsiella pneumoniae*, while the most common Gram-positive one is *Staphylococcus*. Studies have shown that transplant patients with bacterial infections had an increased one-year mortality [94].

### 6.5. Neoplasms

Neoplasms are an important cause of late mortality after HTx. The most likely explanation is the need for immunosuppression, which makes the patient more vulnerable to the carcinogenesis that certain viruses such as EBV, human herpesvirus 8, human papillomavirus, and hepatitis B, C can cause. It is estimated that >10% of transplanted adults will develop malignancy within 5 years [95]. Skin cancers and some rare neoplasms show the greatest increase [96]. Increased risk factors are male sex, old age, white race, and increased duration of administration of intense immunosuppressive therapy [97]. Studies have shown that switching from calcineurin inhibitors to mTOR reduces the chance of cancer [98,99].

### 6.6. Retransplantations

A small number of patients will be retransplanted. This mainly occurs in young patients between 19 and 40 years old who experience severe graft failure due to rejection, graft vasculopathy, and primary graft failure. They are usually patients with a severe clinical picture with an unmet need to support circulation with vasoconstrictor drugs or devices and hemodialysis. Survival rates are poor. Risk factors for survival are the use of LVAD, older age, increased ischemic time, and primary graft failure. For now, due to the international shortage of grafts, this specific procedure is limited to selected cases [100,101].

## 7. Future Perspectives

Organs from genetically modified pigs have been the main focus of research in xenotransplantation. The world’s first porcine-to-human xenotransplantation was performed at the University of Maryland School of Medicine (Baltimore, MD, USA), where a genetically modified pig heart was successfully transplanted into a 57-year-old man with non-ischemic cardiomyopathy [102,103]. The historic operation overcame the main obstacle (i.e., hyperacute immune rejection) and achieved a good short-term outcome. However, the patient died two months after the transplant surgery. A second porcine-to-human xenotransplantation was performed on a 58-year-old patient with heart failure, who died nearly six weeks after the highly experimental surgery.

**Figure 1:**
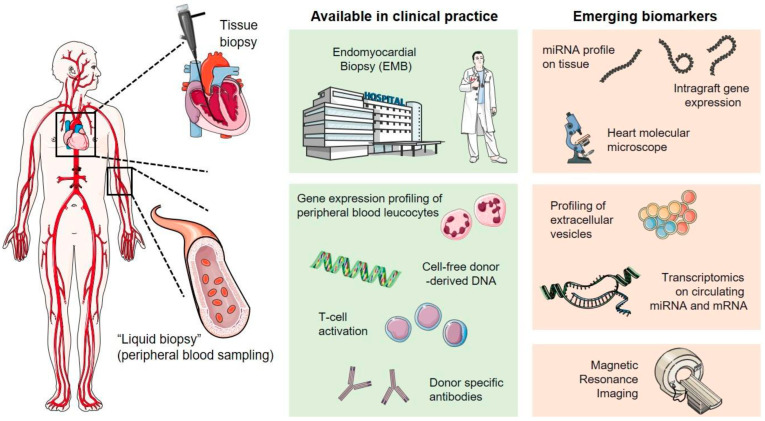
Invasive and non-invasive approaches in cardiac allograft rejection monitoring. Adapted from Giarraputo, A. et al. Biomolecules 2021, 11, 201 [108].

Advances in the pre-transplantation field, such as the inclusion of hepatitis C donors or donation after circulatory death and ex situ donation, focus on the expansion of the organ donor pool [104,105,106,107]. Furthermore, advances after HTx include novel monitoring for acute cellular rejection (Figure 1) [108] and antibody-mediated rejection (i.e., cell-free DNA, gene expression profiling, and micro RNA molecular microscopy) [109]. Pharmacogenomics, proteomics, and metabolomics are promising techniques for identifying biomarkers to assist in the diagnosis and management of post-transplant complications [110]. Advances in donor heart preservation and transportation (i.e., ex vivo perfusion) aim to minimize ischemic injury to the donor’s heart [105]. Lastly, novel immunosuppression therapies such as daratumumab, belatacept, IL 6 directed therapy, and IgG endopeptidase show promising results [104].

## 8. Conclusions

HTx is the final therapeutic option for patients with advanced heart failure. Several factors determine the outcome of HTx, such as ABO and HLA compatibility, graft size, ischemic time, and age. Active infection, peripheral vascular disease, malignancies, and increased BMI are frequent contraindications. The major complications of HTx include graft rejection, graft angiopathy, primary graft failure, infection, neoplasms, and retransplantation. Advances in the field of HTx encompass novel monitoring for acute cellular rejection and antibody-mediated rejection. Omics technology may assist in the diagnosis and management of post-transplant complications.

## Figures and Tables

**Table 1:** Main indications for heart transplantation.

1. Advanced heart failure: Cardiogenic shock with the need for circulatory support with devices or the need for continuous administration of inotropes; Use of long-term circulatory support devices; NYHA III–IV, despite optimally tolerated therapy and application of resynchronization therapies; Multiple episodes of fluid retention leading to pulmonary congestion or significant peripheral edema requiring high-dose diuretics or decreased cardiac output at rest requiring inotropic and/or vasoconstrictor drugs leading to >1 unscheduled emergency department visit or hospitalization in a year; Severe cardiac dysfunction with LVEF < 30% or right ventricular dysfunction or inoperable valvular diseases or elevated NT-proBNP values and evidence of severe diastolic dysfunction (ultrasound, cardiac catheterization), or structural abnormalities according to the definitions for HFrEF, HfpEF.
2. Intolerance to mild exercise with peak VO2 < 10 mL/kg/min
3. Recurrent life-threatening ventricular arrhythmias despite ICD use or ablation therapy
4. End-stage heart failure due to congenital defects without evidence of pulmonary hypertension
5. Resistant angina without further possibility of pharmaceutical or surgical treatment

Abbreviations: NYHA, New York Heart Association; LVEF, Left Ventricular Ejection Fraction; HFrEF, Heart Failure with reduced Ejection Fraction; HFpEF, Heart Failure with preserved Ejection Fraction; ICD, Implantable Cardioverter Defibrillator.

**Table 2:** Main contraindications for heart transplantation.

1. Active serious infection, except LVAD device infection, which is an indication
2. Severe peripheral vascular disease with reduced possibility of recovery and no possibility of revascularization
3. Medically irreversible pulmonary hypertension (PASP > 60 mmHg, PVR > 5WU, TPG > 15 mmHg)
4. Malignancies with a poor prognosis
5. Irreversible liver damage or kidney damage (eGFR < 30 mL/min/1.73 m^2^)
6. Systemic disease involving multiple organs
7. Abuse of alcohol or addictive substances
8. Poor social support with inability to care in the period after surgery
9. Serious neurological diseases
10. Active pulmonary embolism
11. Frailty (>10 lbs weight loss, fatigue, muscle cachexia)
12. ΒΜΙ > 35 kg/m^2^
13. Diabetes with HbA1c > 7.5% despite optimal medication and target organ complications excluding retinopathy [63,64]

Abbreviations: LVAD, Left Ventricular Assist Device; PASP, Pulmonary Artery Systolic Pressure; PVR, Pulmonary Vascular Resistance; TPG, Transpulmonary Pressure Gradient; eGFR, estimated Glomerular Filtration Rate; BMI, Body Mass Index; HbA1C, hemoglobin A1C.

**Table 3:** ISHLT classification of vasculopathy.

1. Grade 0: 1. No alterations of the vessels are observed
2. Grade I: Left main stenosis < 50% or primary vessel stenosis < 70% or any branch stenosis < 70%, without graft dysfunction
3. Grade II : Left main stenosis < 50% or single primary vessel stenosis > 70% or isolated branch stenosis > 70% in 2 systems, without graft dysfunction
4. Grade III: Left main stenosis ≥ 50% or stenosis > 70% in two or more primary vessels or isolated branch stenosis > 70% in three systems or milder disease with signs of graft dysfunction (Left Ventricle Ejection Fraction < 45%, Restrictive Pathology with E/A > 2, Deceleration Time < 150 ms, Isovolumetric Relaxation Time < 60 ms, Right Heart Catheterization with Right Atrial Pressure > 12 mmHg, Pulmonary Capillary Wedge Pressure > 25 mmHg and Cardiac Index < 2 L/min/m^2^

**Table 4:** ISHLT criteria for grading the severity of primary graft failure.

**For the left ventricle**
1. Mild: One of the following criteria should be met: Left Ventrical Ejection Fraction < 40% Right Atrial Pressure > 15 mmHg, Pulmonary Capillary Wedge Pressure > 16 mmHg, Cardiac Index < 2 L/min/m^2^ with duration > 1 h requiring low-dose inotropes
2. Moderate: At least one criteria from both groups should be met: One of the criteria for mild disease Need for administration of inotropes in increased doses, placement of intra-aortic balloon
3. Severe: Need to use circulatory support devices excluding intra-aortic balloon
**For the right ventricle**
Either criteria I and II or III alone must be met: I. Right Atrial Pressure > 15 mmHg, Pulmonary Capillary Wedge Pressure > 16 mmHg, Cardiac Index < 2 L/min/m^2^ II. Transpulmonary Pressure Gradient < 15 mmHg and/or Pulmonary Artery Systolic Pressure < 50 mmHg III. Need for RVAD [85]

Abbreviation: RVAD, Right Ventricular Assist Device.

## Data Availability

Not applicable.
